# A Comparative Evaluation of Hydroxyapatite Crystals and Glutaraldehyde as Agents for Pulpotomy in Deciduous Molars

**DOI:** 10.5005/jp-journals-10005-1035

**Published:** 2009-04-26

**Authors:** Vivek Kumar Adlakha, Preetika Chandna, JL Joshi, AM Thomas, Namita Singh

**Affiliations:** 1 Lecturer, Department of Pedodontics and Preventive Dentistry, Subharti Dental College, Meerut, Uttar Pradesh, India; 2 Lecturer, Department of Pedodontics and Preventive Dentistry, Subharti Dental College, Meerut, Uttar Pradesh, India; 3 Former Principal, Christian Dental College, Ludhiana, Punjab, India; 4 Principal, Christian Dental College, Ludhiana; Professor and Head, Department of Pedodontics and Preventive Dentistry Christian Dental College, Ludhiana, Punjab, India; 5 Professor, Department of Pedodontics and Preventive Dentistry, Christian Dental College, Ludhiana, Punjab, India

**Keywords:** Pulpotomy, hydroxyapatite crystals, glutaraldehyde, deciduous molars.

## Abstract

*Purpose:* To evaluate and compare clinically and radiographically use of hydroxyapatite crystals and 2% glutaraldehyde as a pulpotomy agent.

*
Method:* Thirty deciduous molars were treated with pulpotomy using hydroxyapatite crystals and 2% glutaraldehyde.

*
Results:* Clinical and radiographic findings were observed at three months and six months. The success rate was found to be 100% clinically and 80.33% radiographically in the hydroxyapatite crystals group and 100% clinically and radiographically in the glutaraldehyde group.

*
Clinical significance:* The results of this study revealed that hydroxyapatite crystals is a potential pulpotomy agent for deciduous molars.

## INTRODUCTION

The pulp in primary teeth has a high potential for repair because of a high degree of cellularity and vascularity in this tissue.^1^ Further, the young pulp lends itself most readily to procedures concerned with preservation of pulp vitality such as pulpotomy.^2^ The rationale for the pulpotomy procedure is that the radicular pulp tissue is healthy and capable of healing after surgical amputation of the affected or the infected coronal pulp. Thus, pulpotomy helps to maintain the primary dentition in an intact state until the normal exfoliation occurs-a major goal of pediatric dentistry.

There is ample information about pulpotomy in deciduous molars using formocresol, glutaraldehyde, electrosurgery, ferric sulphate, calcium hydroxide, etc. The vital pulpotomy process using formocresol has been widely accepted in primary tooth pulp therapy because of its simplicity and good prognosis.^3^ However, much concern has arisen over the mutagenic and carcinogenic potential of formaldehyde containing products, the cytotoxic effects of formocresol and the possible diffusion into the surrounding and systemic tissues.^4^ In order to avoid the possible harmful effects of formocresol and other pulpotomy agents, an ideal agent for vital pulpotomy procedure is being sought.

In 1976, Dankert, s’Gravenmade and Wemes reported the advantages of glutaraldehyde as an intracanal medicament during endodontic therapy.^5^ Ample evidence has accumulated overtime, which has led investigators to suggest that glutaraldehyde should replace formocresol as the medicament of choice for chemical pulpotomy procedures on primary teeth. Numerous studies have shown that application of 2 to 5% aqueous glutaraldehyde produces surface fixation of the underlying pulpal tissue with limited depth of penetration.^6^-^9^ Glutaraldehyde has more stable interactions with proteins rather than formocresol, as it has two functional aldehyde groups and this accounts for its powerful bactericidal activity.^7^ Thus, glutaraldehyde was chosen as the standard of comparison in this study.

In our endeavour to find an ideal pulpotomy agent, the use of hydroxyapatite crystals was assessed for its regenerative potential. Hydroxyapatite has been shown to be an extremely biocompatible material for soft tissues and bone.^10^ It has been reported to be effective in alveolar ridge augmentation,^10^ healing of periodontal bone defects,^11^ osseointegration of titanium implants^12^ and direct pulp capping.^13^ Hydroxyapatite, which is the main constituent of dental hard tissues, may immediately provide an artificial barrier. Despite the putative abilities of hydroxyapatite to be osteoconductive, osteogenic and dentinogenic,^13^ little research has been done with this material as a pulp healing agent.

The present study was undertaken to evaluate and compare clinically and radiographically the use of hydroxyapatite crystals and glutaraldehyde as pulpotomy agents.

## MATERIALS

The present study was conducted in the Department of Pedodontics and Preventive Dentistry at Christian Dental College, CMC, Ludhiana-141008, Punjab, India. The permission of the ethical committee of the institute was obtained prior to the start of the study.

Thirty patients aged between 4 and10 years attending the Out Patient Clinic of the Department of Pedodontics and Preventive Dentistry at Christian Dental College, CMC, Ludhiana-141008, Punjab, India were selected for the study. A total of thirty deciduous molars were treated.

The teeth indicated for pulpotomy were assessed, the procedures/techniques performed by a single clinician and evaluated after three to six months follow-up period. The criteria for the selection of teeth included in the study are given in Table 1.

Prior to the treatment, the procedure was explained to the parents of the children involved in the study and their informed consent as approved by the head of the institution was obtained.

**Fig. 1.**
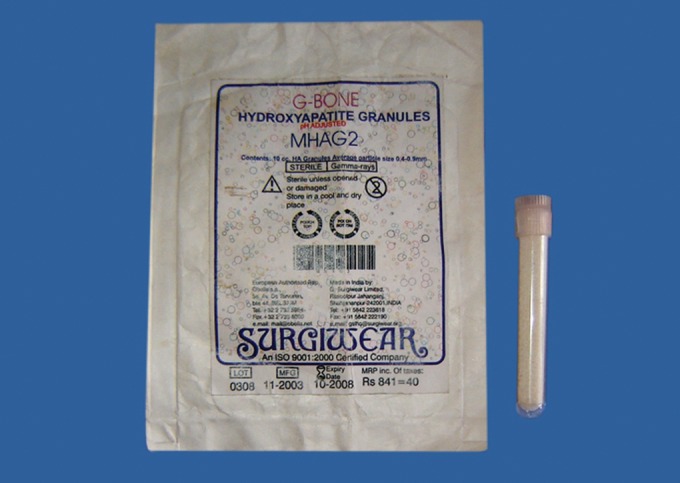
Hydroxyapatite crystals used in the study (300-400 µm)

**Fig. 2.**
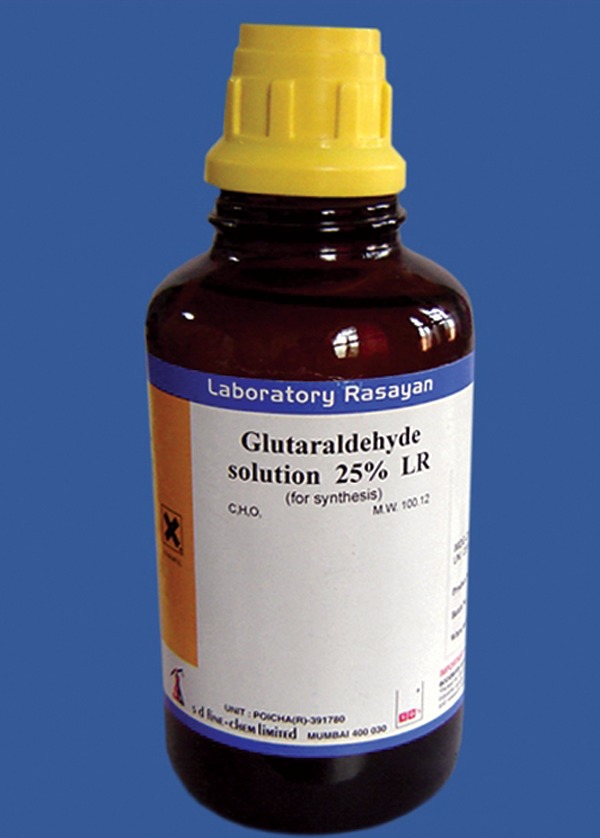
Glutaraldehyde used in the study

Hydroxyapatite crystals with an average particle size of 300-400 µm (G-Bone Hydroxyapatite granules, Surgiwear) were used for the study (Fig.1). The hydroxyapatite crystals were mixed with sterile physiological saline solution to form a paste prior to its application (Fig. 2).

**Fig. 3.**
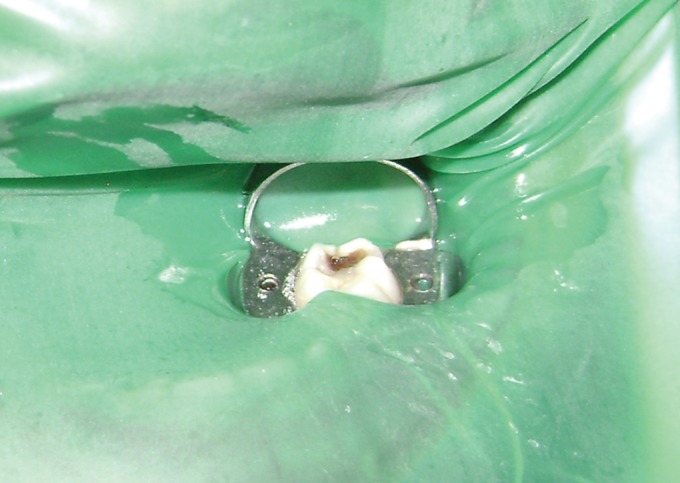
Tooth isolated with rubber dam for hydroxyapatite pulpotomy

A 25% stock solution of glutaraldehyde (s. d. fine-chem limited) (Fig. 3) was used. This solution was diluted with distilled water and 0.2M phosphate buffer to make 2% buffered solution of glutaraldehyde (pH-7.2).14 This solution was kept under refrigeration.

**Table 1:** Table 1: Selection criteria

*S. No.*		*Criteria for the selection of teeth*		*Presence /Absence*
1.		Patient should be within the age group of 4-10 years		✔
2.		Carious/mechanical pulp exposure		✔
3.		There should be no furcation involvement		✔
4.		There should be no clinical or radiographic sign of pulp pathoses		✔
5.		There should be a possibility of proper restoration of the tooth after the procedure		✔
6.		Hemostasis should be easily achievable with a sterile cotton pellet after pulp amputation		✔

## METHODS

**Fig. 4.**
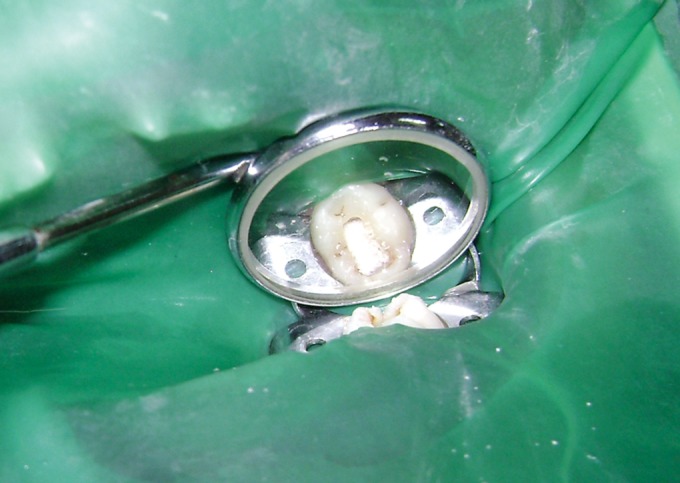
Hydroxyapatite crystals applied to amputated pulp

**Fig. 5.**
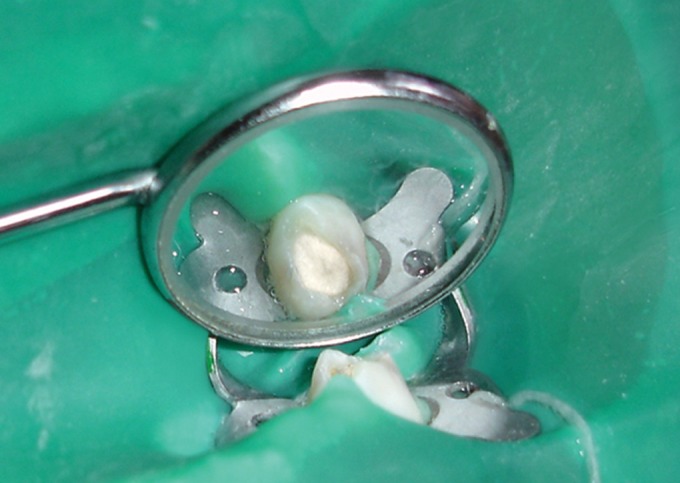
Two percent glutaraldehyde applied to amputated pulp

Thirty largely intact primary molars were selected for the study with the above mentioned inclusion criteria (Table 1). The procedure was carried out step-by-step in one-visit as follows:

1. Under local anesthesia, rubber dam was used to isolate the tooth (Figs 4 and 5).
2. Establishment of cavity outline form was done and all marginal caries was removed before the pulp was exposed.
3. Exposure of the coronal pulp was carried out with a round bur.
4. The access cavity was enlarged to the limit of the pulp horns to simplify coronal pulp removal.
5. The coronal pulp was removed with a sterile sharp spoon excavator. The pulp was amputated at the entrance to the root canal.
6. The pulp chamber was irrigated with sterile saline to prevent dentinal chips from being forced into the radicular pulp.
7. Following irrigation, sterile cotton pellets were applied to the amputated pulp stumps to aid in hemostasis.

After this standardized technique, the selected teeth were randomly divided into two groups consisting of 15 primary molars each:

- Hydroxyapatite crystals group; and
- Glutaraldehyde group.

### Hydroxyapatite Crystals Group (Figs 6 and 7)

1. All the primary molars under study were treated with a paste of hydroxyapatite crystals (mixed in sterile physiological saline solution) such that a layer of the paste covers the floor of the coronal pulp chamber.
2. Zinc oxide eugenol base was then placed over the pulp stumps. Subsequently, the tooth was restored with silver amalgam.

### Glutaraldehyde Group (Figs 8 and 9)

1. All the primary molars under study in this group were treated with a cotton pellet moistened with 2% glutaraldehyde.
2. The moistened cotton pellet was placed over the radicular pulp for 5 minutes and then removed.
3. Zinc oxide eugenol base was then placed over the pulp stumps. Subsequently, the tooth was restored with silver amalgam.

**Fig. 6.**
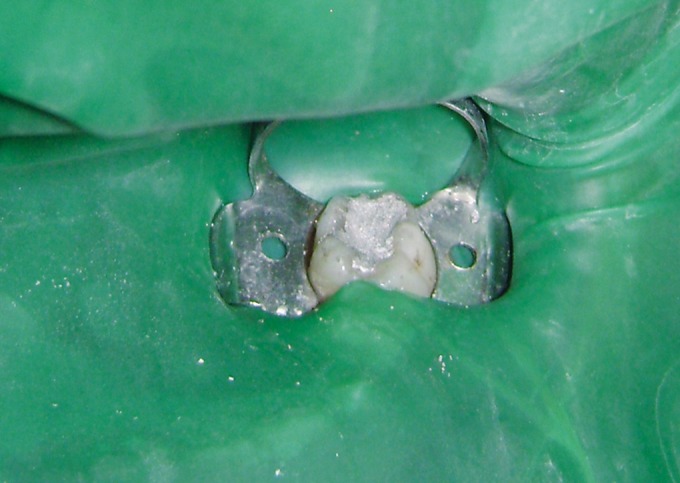
Hydroxyapatite pulpotomized tooth restored with silver amalgam

**Fig. 7.**
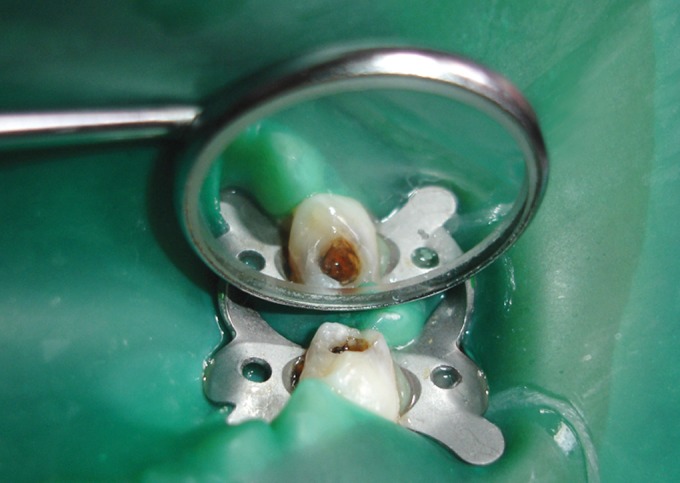
Tooth isolated with rubber dam for glutaraldehyde pulpotomy

**Fig. 8.**
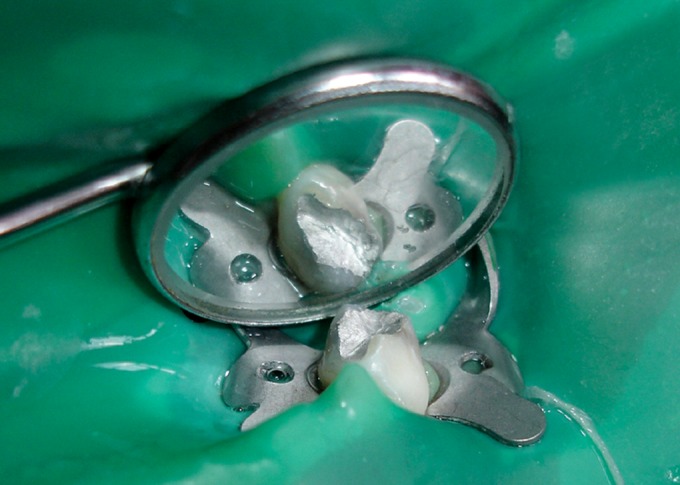
Glutaraldehyde pulpotomized tooth restored with silver amalgam

**Fig. 9.**
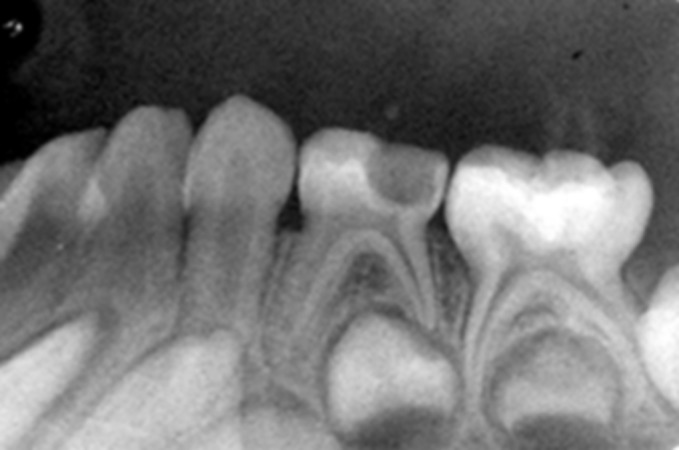
Preoperative IOPA radiograph of hydroxyapatite pulpotomized tooth

**Fig. 10.**
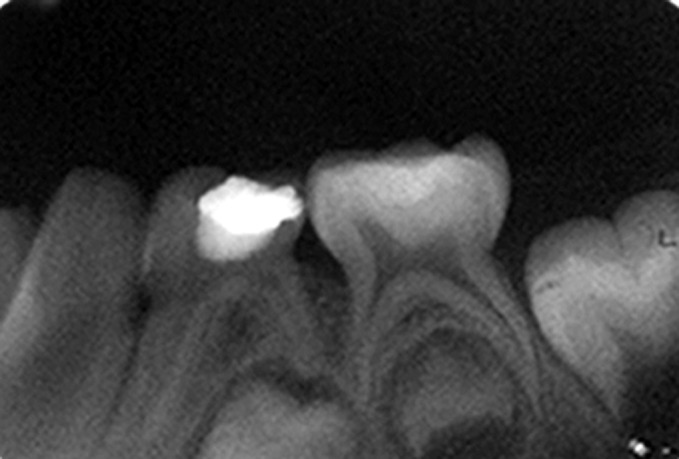
Immediate postoperative IOPA radiograph of hydroxyapatite pulpotomized tooth

**Fig. 11.**
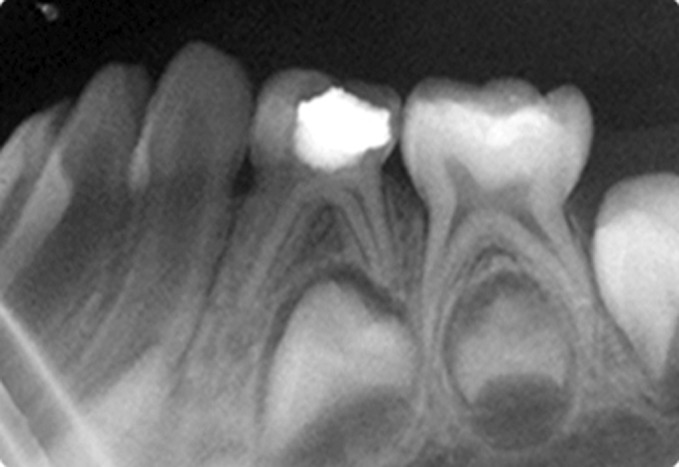
IOPA radiograph at 3 months recall of hydroxyapatite pulpotomized tooth

**Fig. 12.**
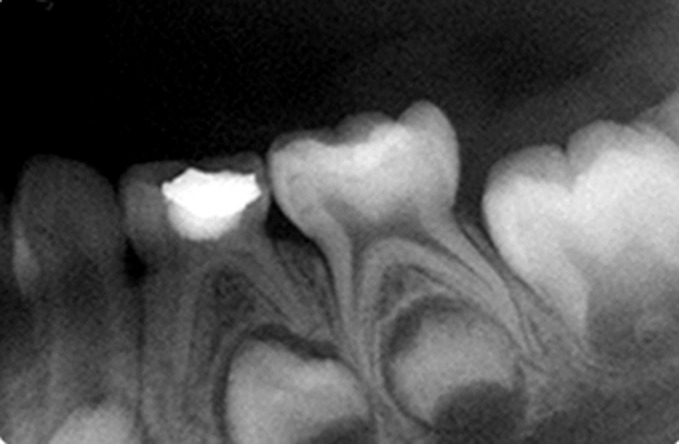
IOPA radiograph at 6 months recall of hydroxyapatite pulpotomized tooth

**Fig. 13.**
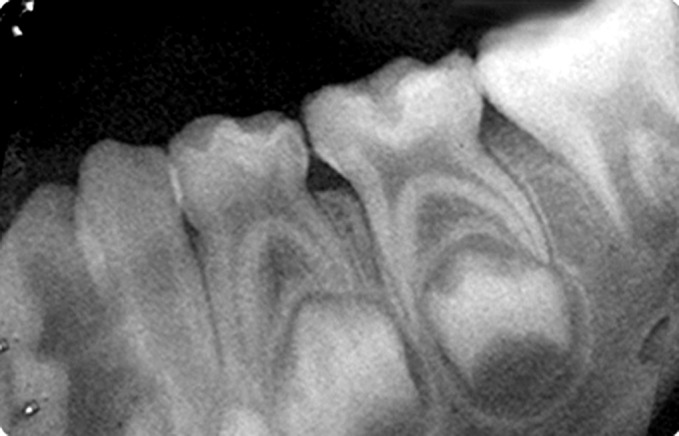
Preoperative IOPA radiograph of glutaraldehyde pulpotomized tooth

**Fig. 14.**
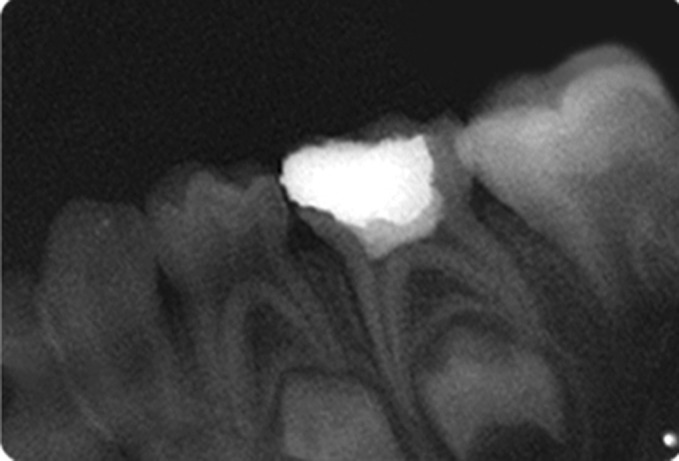
Immediate postoperative IOPA radiograph of glutaraldehyde pulpotomized tooth

**Fig. 15.**
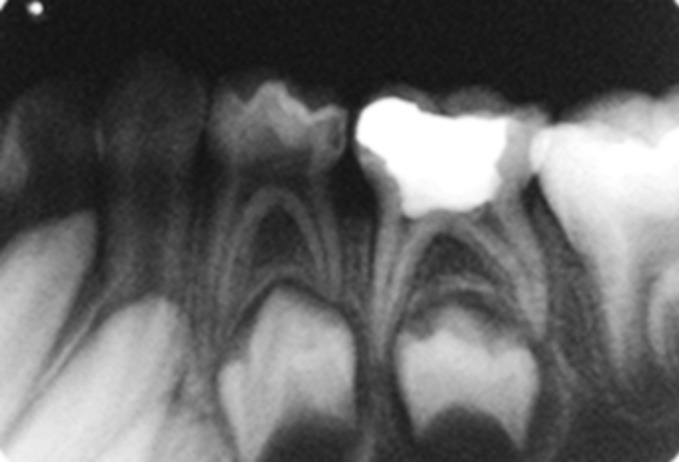
IOPA radiograph at 3 months recall of glutaraldehyde pulpotomized tooth

**Fig. 16.**
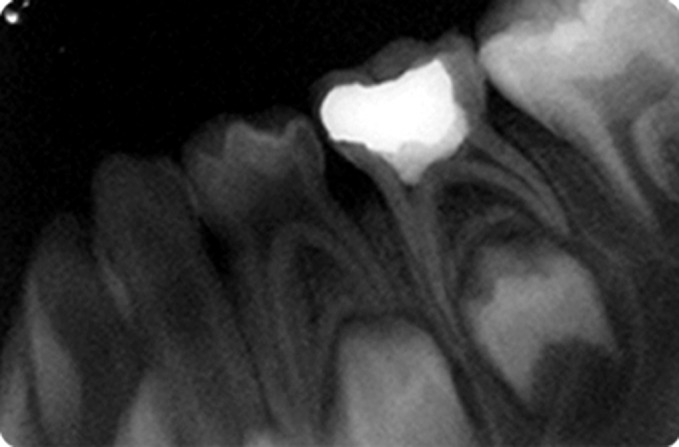
IOPA radiograph at 6 months recall of glutaraldehyde pulpotomized tooth

An intraoral periapical radiograph was taken after the procedure. The children under study were recalled for clinical and radiographic examination at follow-up of 3 months and 6 months (Figs 10 to 16).

Clinical assessment at the follow-up examination (Table 2).

Radiographic assessment at the follow-up examination (Table 3).

The treatment was regarded as a failure when one or more of the above-mentioned signs and symptoms were present, but pulp calcification and absence of dentinal bridge were not regarded as a failure. The data obtained was tabulated and statistical analysis done. The differences in the clinical and the radiographic success among the two groups were statistically analyzed by Chi-square test.

**Table 2:** Table 2: Clinical assessment

*S. No.*		*Clinical evaluation criteria*		*Yes*		*No*
1.		Mobility of the tooth				
2.		Initiation of pain				
3.		Presence of swelling				
4.		Development of sinus in the surrounding tissues				

**Table 3:** Table 3: Radiographic assessment

*S. No.*		*Radiographic evaluation criteria*		*Yes*		*No*
1.		Area of rarefaction				
2.		Internal resorption				
3.		Crypt surrounding the succedaneous tooth not intact				
4.		Radiolucency at the periapical region				
5.		Canal calcification				

## RESULTS

The data obtained was tabulated at three months and six months intervals both clinically and radiographically with respect to individual criteria.

Table 4 illustrates the results of clinical evaluation of pulpotomized primary molars using hydroxyapatite crystals and glutaraldehyde. The follow-up evaluation revealed 100% clinical success in both the groups.

Table 5 illustrates the results of radiographic evaluation of pulpotomized primary molars using hydroxyapatite crystals and glutaraldehyde. The follow-up examination revealed 100% radiographic success in the glutaraldehyde group. Whereas in the hydroxyapatite crystals group, 12 out of 15 primary molars (80.33%) showed radiographic success at 6 months follow-up. There was 1 case of failure at 3 months and 2 more cases of failure at 6 months interval.

## COMPARISON OF CLINICAL RESULTS

Chi-square test was not deemed necessary between the two groups for clinical evaluation because of the 100% results obtained in both the groups.

**Table 4:** Table 4: Clinical assessment

	*Criteria*		*Follow-up period*		*Clinical success*						
	
					*Hydroxyapatite crystals*				*Glutaraldehyde*		
					*No.*		*%*		*No.*		*%*
	Primary molars without mobility		3m		15/15		100%		15/15		100%
			6m		15/15		100%		15/15		100%
	Primary molars without pain		3m		15/15		100%		15/15		100%
			6m		15/15		100%		15/15		100%
	Primary molars without sinus		3m		15/15		100%		15/15		100%
			6m		15/15		100%		15/15		100%
	Primary molars without swelling		3m		15/15		100%		15/15		100%
			6m		15/15		100%		15/15		100%

**Table 5:** Table 5: Radiographic assessment

	*Criteria*		*Follow-up period*		*Radiographic success*						
					*Hydroxyapatite crystals*				*Glutaraldehyde*		
					*No.*		*%*		*No.*		*%*
	Primary molars without area of rarefaction		3m		15/15		100%		15/15		100%
			6m		12/15		80.33%		12/15		100%
	Primary molars without internal resorption		3m		14/15		93.33%		14/15		100%
			6m		13/15		86.67%*		13/15		100%
	Primary molars without intact crypt of succedaneous tooth		3m		15/15		100%		15/15		100%
			6m		15/15		100%		15/15		100%
	Primary molars without periapical radiolucency		3m		15/15		100%		15/15		100%
			6m		15/15		100%		15/15		100%
	Primary molars without periapical canal calcification		3m		15/15		100%		15/15		100%
			6m		14/15		93.33%		14/15		100%
* These two cases showed concomitant area of rarefaction											

### Comparison of Radiographic Results

The 3 months radiographic comparison of *Hydroxyapatite Crystals Group versus Glutaraldehyde Group* gave a chisquare value Χ^2^= 7.06 (p ≤0.05). This value shows a statistically significant difference in the success rates between hydroxyapatite crystals group with glutaraldehyde group.

The 6 months radiographic comparison between *Glutaraldehyde Group and Hydroxyapatite Crystals Group* gave a chi-square value Χ^2^ = 21.85 (p ≤0.001). Statistically, this value shows a highly significant difference in the success rates between these groups.

## DISCUSSION

Development of newer materials that are biocompatible and have good results in the vital pulp therapy procedure like pulpotomy are being tried. In our study, hydroxyapatite crystals and glutaraldehyde were used for the pulpotomy procedure in the cariously exposed deciduous molars.

Wemes JC and s’Gravenmade EJ^15^ have proposed glutaraldehyde as a fixative for pulp in dentistry. Experimental^16^- ^18^ and clinical^19^-^21^ studies have been performed with this drug, seeking satisfactory clinical action with minimal side effects. The properties of glutaraldehyde which make it a potential agent for pulpotomy procedure and also an alternative to the preferred formocresol, are its superior fixative properties, self-limiting penetration, low antigenicity and low toxicity. Hence, this medicament was used in our comparative evaluation study.

The rationale for the use of hydroxyapatite crystals in our study was simple. This was done because the mineral content of bone and teeth is a calcium phosphate salt, hydroxyapatite and perhaps with its biocompatibility, osteoconductive, and dentinogenic properties would be a potential medicament for pulpotomy procedure. Special care was taken in choosing the teeth for this study to assure similarity in amount of caries involvement and presumably, pulpal involvement.

The clinical (Table 4) and radiographic success rate (Table 5) was 100% in our study for the glutaraldehyde group. This confirmed the results of previous studies that glutaraldehyde is a satisfactory pulp medicament in human primary teeth as the reported success rate of the pulpotomies were higher than 90%. Prakash C et al^22^ reported 100% clinical and radiographic success following glutaraldehyde pulpotomy in 6 months evaluation. Fuks AB et al^23^ reported the successful use of 2% buffered glutaraldehyde solution in pulpotomies of primary molars. Shumayrikh NM and Adenobi JO^21^ reported a 96.5% and 75.8% clinical and radiographic success rate, respectively, using glutaraldehyde with ZOE base in 12 months follow-up period.

Glutaraldehyde has long been considered as an excellent fixative agent for biological purposes. Since glutaraldehyde is a bifunctional reagent, it has the ability to form strong intra- and intermolecular protein bonds, leading to fixation.^24^ This rapid and more complete fixation as well as stability is advantageous in clinical situations. Moreover, glutaraldehyde apparently causes no inflammation when used in the treatment of vital and non-vital pulps of humans.^25^

There has been considerable research dealing with the diffusibility of pulp medicaments from the confines of the tooth. Glutaraldehyde does not exhibit this ability to leach out of the tooth according to data by Dankert J et al.^5^

The radiographic evaluation of glutaraldehyde group showed one case with pulp canal calcification. This was not considered as a radiographic failure. This calcific metamorphosis is apparently the result of odontoblastic activity due to irritation caused by the fixative on chronically inflamed radicular pulp.^26^

Conversely, long-term follow-up studies have not shown similar success rates. Fuks AB et al^23^ reported a 90.4% success rate after 12 months, which dropped down to 82% at 25 months recall. Similarly, Tsai TP et al^27^ obtained a 98% clinical success rate but when combined with radiographic evaluation, the average success rate was 78.7% after 36 months.

In the hydroxyapatite crystals group, the treatment was clinically successful for all the 15 teeth in the 6 months follow-up period (Table 4). However, the radiographic findings (Table 5) showed a success of 93.33% at a 3 months follow-up period (with 1 case of failure) and a success of 80.33% at a 6 months follow-up period (with 3 cases of failure), according to the criteria used in this study.

One case showed internal resorption at 3 months’ followup but no clinical signs of failure were evident. The internal resorption/area of rarefaction seen in our study might have been influenced by the particle size and shape. Stanley HR^28^ pointed out that inflammation seen in the capped teeth can be because of the particles of the capping agent which could further lead to necrosis. As Misiek DJ et al^29^ described that the irregularly shaped, sharp edged particles promoted more inflammatory response. This can lead to resorption of dentin and the surrounding bone.

On subsequent radiographic evaluation at 6 months, this case showed furcation involvement and an area of rarefaction and was judged as radiographic failure. However, the tooth remained clinically sound. The other two cases showed internal root resorption/area of rarefaction at 6 months follow-up. For the latter two cases, the radiographs at 3 months follow-up showed no abnormal radiographic findings. The three cases of radiographic failure may have been due to the application of hydroxyapatite crystals on the previously inflamed radicular pulp and/or because of the zinc oxide eugenol base applied over the hydroxyapatite crystals.

The 100% clinical success during the 6 months follow up is in accordance with the study by Frank RM et al (2001).^30^ Frank RM et al carried out pulp capping with microsized hydroxyapatite in premolars and found healing was uneventful clinically after a period of 6 months.

There are three available forms of hydroxyapatite; a dense, particulate, non-resorbable form; a porous form derived from the exoskeleton of coral; and a resorbable nonceramic hydroxyapatite.^31^ In the present study, hydroxyapatite with particle size ranging from 0.4-0.9 mm (400-900 µm) was used. The pH of the material is preadjusted to neutral pH. Higashi T and Okamoto H^32^,^33^ in two separate studies found the influence of particle size of hydroxyapatite on cell proliferation of cultured fibroblasts and on the formation of a hard tissue barrier in amputated dental pulp. After experimental pulpotomy, the authors found two types of hard tissue barriers that were formed, namely the osteodentin structure and the tubular dentin structure. They also demonstrated that larger particles of hydroxyapatite are biocompatible and small particles are possibly considered by the tissue to be a foreign body and thus rejected. The pulp canal obliteration seen in the pulpotomized teeth could possibly be due to the migration of hydroxyapatite crystals into the radicular pulp.

Reparative dentin is easily identified in histological sections. In the present study, dentin bridge formation was not reported since this was a clinical and radiographic study. Dentin bridge is formed adjacent to exposed pulp and is not always seen in intraoral periapical X-ray as it is very difficult to have radiographic beam perfectly perpendicular to the axis of tooth and at exposed radicular pulp at the same time. Hence, dentin bridge formation was not used as one of the criteria for radiographic success.

Through the present study, an attempt was made to use hydroxyapatite crystals as a pulpotomy agent for cariously exposed deciduous molars and a comparison was made with 2% glutaraldehyde, clinically and radiographically. Hydroxyapatite crystals provided acceptable success in the study though the glutaraldehyde treated teeth showed a significantly better radiographic success. Further research on a larger sample size, using hydroxyapatite crystals including histologic criteria, are needed to confirm the results of this study.

## CONCLUSION

Hydroxyapatite crystals can be used as a viable material for pulpotomy of cariously exposed deciduous molars.
